# PERM1 regulates genes involved in fatty acid metabolism in the heart by interacting with PPARα and PGC-1α

**DOI:** 10.1038/s41598-022-18885-3

**Published:** 2022-08-26

**Authors:** Chun-yang Huang, Shin-ichi Oka, Xiaoyong Xu, Chian-Feng Chen, Chien-Yi Tung, Ya-Yuan Chang, Youssef Mourad, Omair Vehra, Andreas Ivessa, Ghassan Yehia, Peter Romanienko, Chiao-Po Hsu, Junichi Sadoshima

**Affiliations:** 1 Department of Cell Biology and Molecular Medicine, Rutgers New Jersey Medical School, 185 South Orange Ave., MSB G609, Newark, NJ 07103 USA; 2 Division of Cardiovascular Surgery, Department of Surgery, Taipei Veterans General Hospital, Taipei, Taiwan; 3 Department of Medicine, School of Medicine, National Yang-Ming Chiao-Tung University, Taipei, Taiwan; 4 Department of Cardiology, Ningbo Medical Center Lihuili Hospital, Ningbo, Zhejiang China; 5 Cancer Progression Research Center, National Yang-Ming Chiao-Tung University, Taipei, Taiwan; 6 Genome Editing Core Facility, Rutgers Cancer Institute of New Jersey, New Brunswick, NJ 08901 USA

**Keywords:** Cell biology, Cardiology

## Abstract

PERM1 (PGC-1/ERR-induced regulator in muscle 1) is a muscle-specific protein induced by PGC-1 and ERRs. Previous studies have shown that PERM1 promotes mitochondrial biogenesis and metabolism in cardiomyocytes in vitro. However, the role of endogenous PERM1 in the heart remains to be investigated with loss-of-function studies in vivo. We report the generation and characterization of systemic *Perm1* knockout (KO) mice. The baseline cardiac phenotype of the homozygous *Perm1* KO mice appeared normal. However, RNA-sequencing and unbiased pathway analyses showed that homozygous downregulation of PERM1 leads to downregulation of genes involved in fatty acid and carbohydrate metabolism in the heart. Transcription factor binding site analyses suggested that PPARα and PGC-1α are involved in changes in the gene expression profile. Chromatin immunoprecipitation assays showed that PERM1 interacts with the proximal regions of PPAR response elements (PPREs) in endogenous promoters of genes involved in fatty acid oxidation. Co-immunoprecipitation and reporter gene assays showed that PERM1 promoted transcription via the PPRE, partly in a PPARα and PGC-1α dependent manner. These results suggest that endogenous PERM1 is involved in the transcription of genes involved in fatty acid oxidation through physical interaction with PPARα and PGC-1α in the heart in vivo*.*

## Introduction

PERM1 (PGC-1/ERR-induced regulator in muscle 1) is a muscle-specific protein induced by PGC-1α and ERRα and required to express a subset of genes essential for muscle metabolism^1^. PERM1 acts as a positive regulator of mitochondrial biogenesis and the oxidative capacity in C2C12 cells in vitro and skeletal muscles in vivo and cultured cardiomyocytes in vitro^2,3^*.* Pathway enrichment analyses revealed that downregulation of PERM1 inhibits cellular metabolism in cultured cardiomyocytes^2^. The promoter region of the *Perm1* gene includes a consensus ERR binding sequence and expression of PERM1 is promoted by PGC-1α and ERRα in vitro^2,4,5^. Conversely, PERM1 also enhances transcriptional activity of ERRα and upregulates a known target of ERRα, Ndufv1 in cardiomyocytes^2^. These observations suggest that PERM1 possesses an intimate interaction with PGC-1α and ERRα.

PERM1 is upregulated by exercise in skeletal muscles and in response to cold stress in inguinal white adipocytes^3,6,7^. It is also upregulated in the heart during cardiogenesis and postnatal cardiac growth, whereas it is downregulated during pathological hypertrophy and heart failure^2,8^. PERM1 confers fatigue resistance to skeletal muscle in vivo through increases in mitochondrial content and oxidative capacity and angiogenesis^3^. PERM1 also reduces cellular damage resulting from hypoxia and reoxygenation-induced stress and mitigates cell death of cardiomyocytes in vitro^8^. However, the role of endogenous PERM1 in the pathophysiology of the heart remains unclear due to insufficient characterization of PERM1 loss-of-function mouse models.

Although it has been suggested that there is an intimate interaction between PERM1 and the nuclear PGC-1α-ERRα pathway in the heart and skeletal muscles, the molecular identity of PERM1 remains unclear. Previous studies have shown that PERM1 is located both in the nucleus and the perinuclear space^2,8–10^. PERM1 in the nucleus physically interacts with PGC-1α, thereby regulating nuclear transcription^8^. PERM1 on the outer mitochondrial membrane interacts with intracellular adapter protein ankyrin B, thereby connecting mitochondria with the sarcolemma^10^. Furthermore, it has been proposed that PERM1 located on the outer mitochondrial membrane controls the homeostasis of lipid and amino acid metabolites in mitochondria in the heart^9^. However, how endogenous PERM1 in the nucleus controls transcription of genes involved in mitochondrial biogenesis and oxidative phosphorylation in vivo remains to be clarified, using PERM1 loss-of-function models.

In this study, we conducted an unbiased pathway analysis in order to identify the functionally relevant signaling mechanisms regulated by endogenous PERM1 in the heart and found that PPARα and PGC-1α are the major mechanisms mediating the effect of PERM1 upon metabolism in the heart in vivo. PPARα is a nuclear receptor that induces genes involved in fatty acid uptake and oxidation. Furthermore, PERM1 and PPARα both interact with PGC-1α^8,9^. However, the relationship between PERM1 and PPARα, in particular, whether and how PERM1 and PPARα interact with one another, and how PERM1 affects the function of PPARα remain unclear. Thus, our goal in this study was to elucidate the role of endogenous PERM1 in the heart in vivo, using *Perm1* systemic knockout (KO) mice. We conducted RNA sequencing and bioinformatic analyses and investigated the effect of endogenous PERM1 upon the gene expression profile in the postnatal heart in an unbiased manner. We here report that PPARα plays an important role in mediating the effect of PERM1 upon transcription of genes involved in metabolism in the heart.

## Results

### Generation of systemic *Perm1* knockout (KO) mice

**Figure 1:**
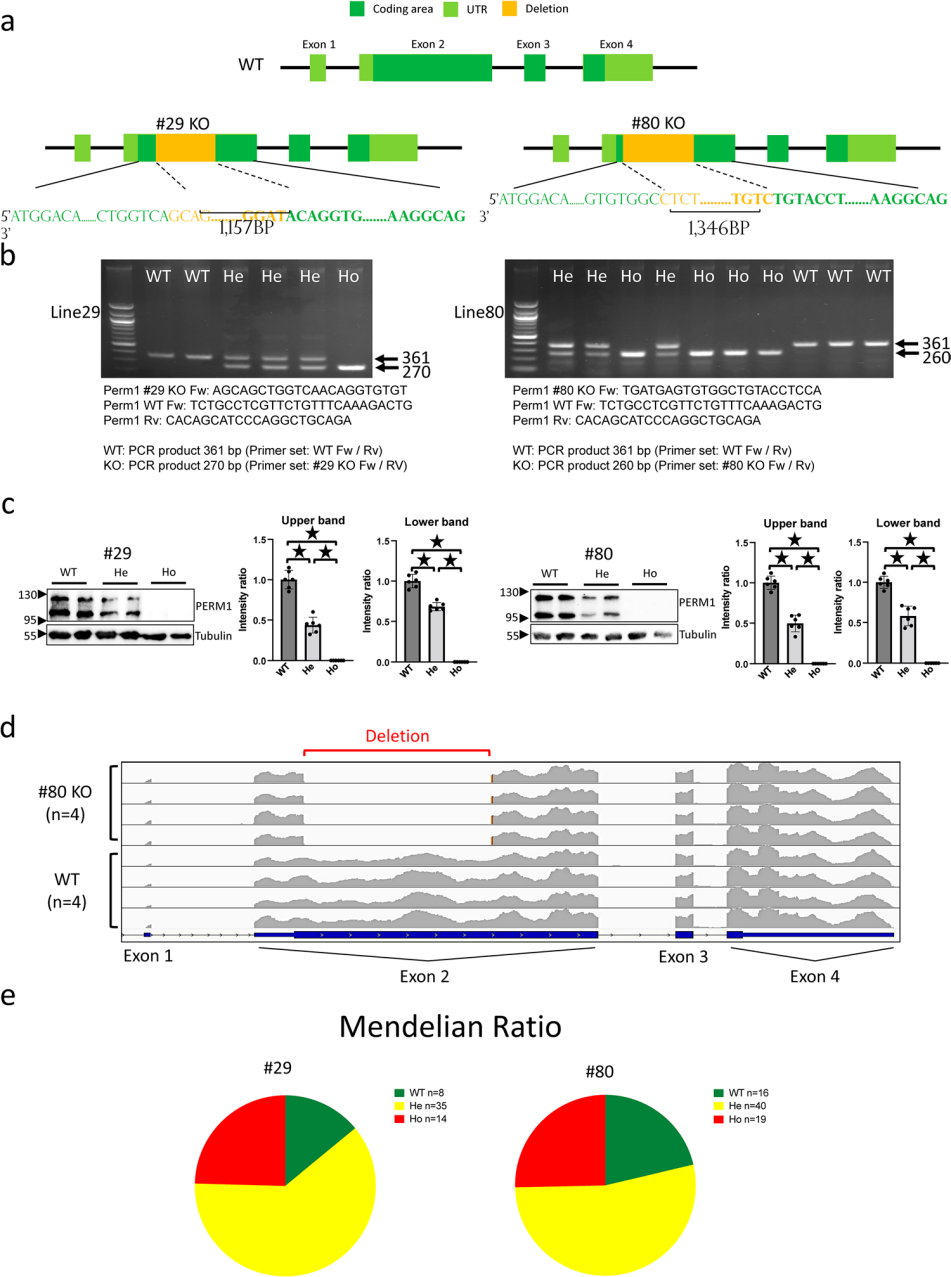
*Perm1* knockouts displayed survival rate, Mendelian rule, and body weight growth similar to those of wild type. (**a**) Schematic representation of genomic DNA deletion in Line 29 and Line 80 *Perm1* KO mice. The deletion sequence was designed in exon 2. (**b**) DNA electrophoresis for genotyping. Left panel shows 361 bp in WT mice and 270 bp in KO for Line 29 mice. Right panel shows 361 bp in WT and 260 bp for Line 80 mice. The primer sequences for genotyping are shown. (**c**) Western blot analysis of PERM1, with densitometry for WT, heterozygous KO (He), and homozygous KO (Ho) shown individually. Left panel: Line 29; Right panel: The statistical significance was determined with Student’s *t* test, *p < 0.05 was considered significant. Line 29: WT (3 male + 3 female); He (3 male + 3 female); Ho (3 male + 3 female). Line 80: WT (3 male + 3 female); He (3 male + 3 female); Ho (3 male + 3 female). (**d**) Exon level visualization revealing *Perm1* mRNA partial exon 2 deletion in homogenous *Perm1* KO mice. WT (2 male + 2 female); KO (2 male + 2 female). (**e**) Upper and lower panels show 2-month survival rates for Line 29 and Line 80 individually. Line 29: WT (4 male + 4 female); He (16 male + 19 female); Ho (6 male + 8 female). Line 80: WT (7 male + 9 female); He (21 male + 19 female); Ho (11 male + 8 female).

The strategy used to generate systemic *Perm1* KO mice is shown in Fig. 1a. We used the CRISPR-Cas9 technique to target Exon 2, where the start codon is located. The deletion in Exon 2 induces a frameshift and a premature stop codon. Since mRNA with an artificial codon is unstable, we expected that the PERM1 protein with a premature stop codon would be degraded. The forward primer for genotyping of the wild type (WT) mice was designed to bind to the deleted sequence. The forward primer for the KO mice was designed to bind before the deleted sequence. The reverse primer was designed to bind after the deleted sequence. DNA gel electrophoresis showed that homo- and heterozygous KO mice were generated in lines 29 and 80 (Fig. 1b). To further confirm that PERM1 is knocked out, heart samples were subjected to Western blot analyses. Two bands, reflecting the alternative start codon usage (Cho et al. JBC 2021), were observed in WT mouse hearts. However, the intensity of the two bands was decreased 40–60% in heterozygous KO mouse hearts and totally abolished in homozygous KO mouse hearts (Fig. 1c), suggesting that PERM1 is not produced after partial deletion of Exon 2. RNA-sequencing analyses of the heart samples obtained from line 80 exhibited a long sequence deletion at Exon 2 in *Perm1* KO mouse hearts compared to in WT mouse hearts (Fig. 1d). WT, heterozygous and homozygous KO mice were born in the expected Mendelian ratio in each line (Fig. 1e). There was no mortality during the initial 60 days in WT, Line 29 *Perm1* KO, or Line 80 *Perm1* KO mice (Supplementary Fig. S1a). Body weights were also similar between WT and both lines of *Perm1* KO mice (Supplementary Fig. S1b).

### *Perm1* knockout does not significantly affect cardiac function at baseline

**Figure 2:**
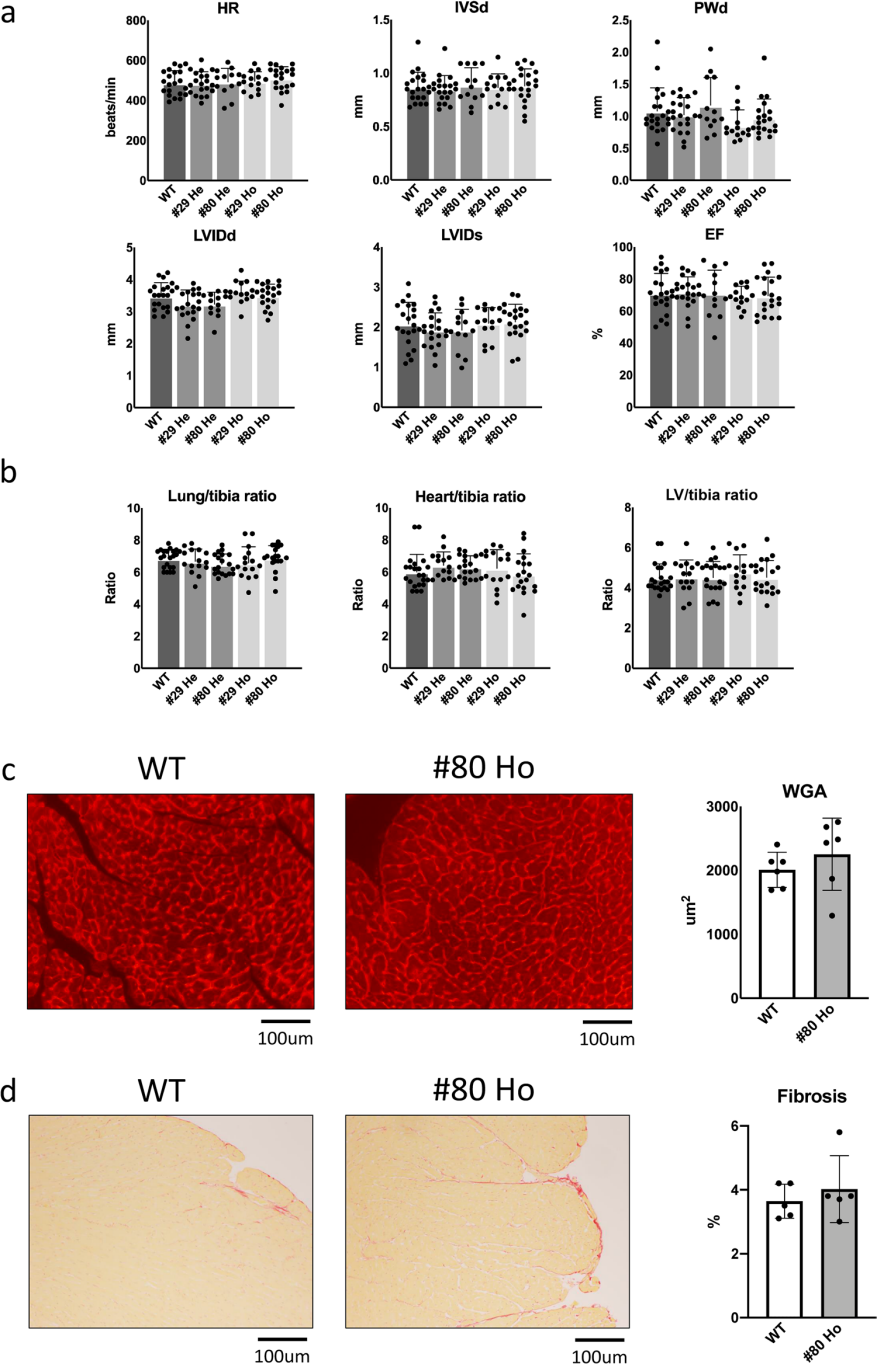
*Perm1* knockout displayed cardiac function similar to that of wild type. (**a**) WT, Line 29 He, Line 80 He, Line 29 Ho, and Line 80 Ho mice at 3 months old were subjected to echocardiographic analyses. Bar graphs show heart rate (HR), IVSd (diastolic interventricular septum thickness), PWd (diastolic posterior wall thickness), LVIDd (end-diastolic LV internal dimension), LVIDs (end-systolic LV internal dimension), and EF (ejection fraction) for WT (10 male + 11 female), Line 29 He (10 male + 10 female), Line 80 He (7 male + 6 female), Line 29 Ho (7 male + 7 female), and Line 80 Ho (10 male + 10 female). (**b**) Lung, heart and LV weight to tibial bone length ratios for WT (11 male + 11 female), Line 29 He (7 male + 8 female), Line 80 He (10 male + 9 female), Line 29 Ho (9 male + 6 female), and Line 80 Ho (9 male + 10 female) after sacrifice at 3 months old. (**c**) WGA histology showing comparison of cardiomyocyte size between WT (3 male + 3 female) and Line 80 Ho (3 male + 3 female) at 3 months old. (**d**) PSR histology showing comparison of the fibrotic area between WT (3 male + 2 female) and Line 80 Ho (3 male + 2 female) at 3 months old. The statistical significance was determined with 1 way ANOVA (**a**,**b**), Student’s *t* test (**c**,**d**), *p < 0.05 was considered significant (**a**–**d**).

Characterization of the baseline cardiac phenotype was conducted using 3-month-old mice. Echocardiographic analyses showed that parameters of cardiac dimensions, including IVSd (diastolic interventricular septum thickness), PWd (diastolic posterior wall thickness), LVIDd (end-diastolic LV internal dimension), LVIDs (end-systolic LV internal dimension), systolic function EF (ejection fraction), and heart rate (HR), are all similar among WT, line 29 *Perm1* (either heterozygous or homozygous) KO, and line 80 *Perm 1* (either heterozygous or homozygous) KO mice (Fig. 2a and Supplementary Table S1). Organ weights, including lung weight/tibial length, heart weight/tibial length and LV weight/tibial length, were also all similar among WT, line 29 *Perm1* (either heterozygous or homozygous) KO, and line 80 *Perm1* (either heterozygous or homozygous) KO mice (Fig. 2b). Likewise, the histologically determined LV cardiomyocyte cross-sectional area and fibrosis area were similar between WT and line 80 *Perm1* homozygous KO mice (Fig. 2c,d). In summary, *Perm1* knockout does not significantly affect cardiac parameters at baseline at 3 months of age.

### PERM1 regulates metabolic genes in cardiomyocytes in vivo

**Figure 3:**
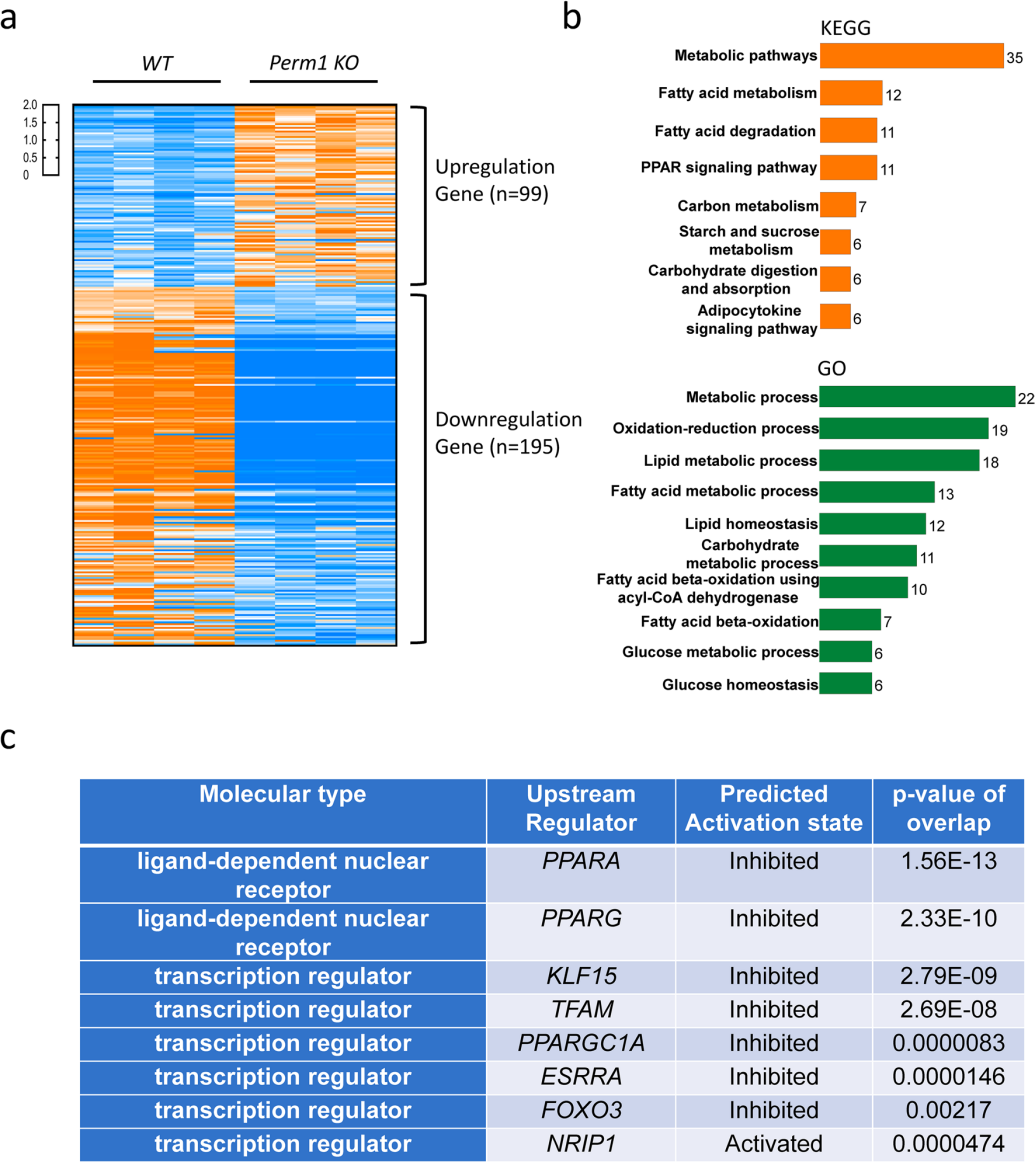
PERM1 regulates genes involved in metabolism in the mouse heart. (**a**) Illumina-based RNA-seq showing genes differentially expressed in *Perm1* KO mice and WT mice, including 99 upregulated genes and 195 downregulated genes (n = 4/group, all male, 2 months old, The statistical significance was determined with Student’s *t* test, *p < 0.05 was considered significant. cantly regulated genes showed that the pathway and protein function affected in *Perm1* KO mice is metabolism. (**c**) Transcription factor analysis by Ingenuity Pathway Analysis (IPA, Qiagen Inc). The significant p-value indicates likely inhibition or activation states of an upstream regulator in *Perm1* KO mice compared with WT mice.

To investigate the effect of endogenous PERM1 downregulation upon mRNA expression in the heart, RNA-sequencing analysis was conducted. Lumina-based RNA-seq analysis of the WT and *Perm1* KO mouse hearts detected 55,451 genes, of which 294 genes were significantly altered by *Perm1* deletion (99 genes were upregulated and 195 genes were downregulated, p < 0.05) (Fig. 3a). Using the DAVID Bioinformatics Resources, the differentially expressed genes in *Perm1* KO heart tissue were analyzed for biological processes based on gene ontology (GO) and Kyoto Encyclopedia of Genes and Genomes (KEGG) pathways. The eight most significantly enriched KEGG pathways and ten most significantly enriched GO biological processes in *Perm 1* KO hearts are shown in Fig. 3b. These results suggest that endogenous PERM1 regulates metabolic pathways, including fatty acid and carbohydrate metabolism, in the heart. Many genes involved in fatty acid metabolism and carbohydrate metabolism are downregulated (Supplementary Tables S2 and S3). Genes involved in the mitochondrial electron transport chain were not altered significantly. Furthermore, ingenuity pathway analysis (IPA) and transcription factor analysis indicated that transcription factors, including PPARA, PPARG, KLF15, TFAM, PPARGC1A, ESRRA and FOXO3, are predicted to be in an inhibition state in *Perm1* KO mice compared to in WT mice. Of note, downregulation of PPARα target genes was the most prominent change in the *Perm1* KO mice. On the other hand, NRIP1 was predicted to be in an activation state (Fig. 3c). Taken together, these results suggest that PERM1 affects metabolic pathways by positively or negatively affecting transcription factors to control genes involved in metabolism in the heart at baseline.

### *Perm1* KO downregulates genes involved in fatty acid and carbohydrate metabolism in the heart

**Figure 4:**
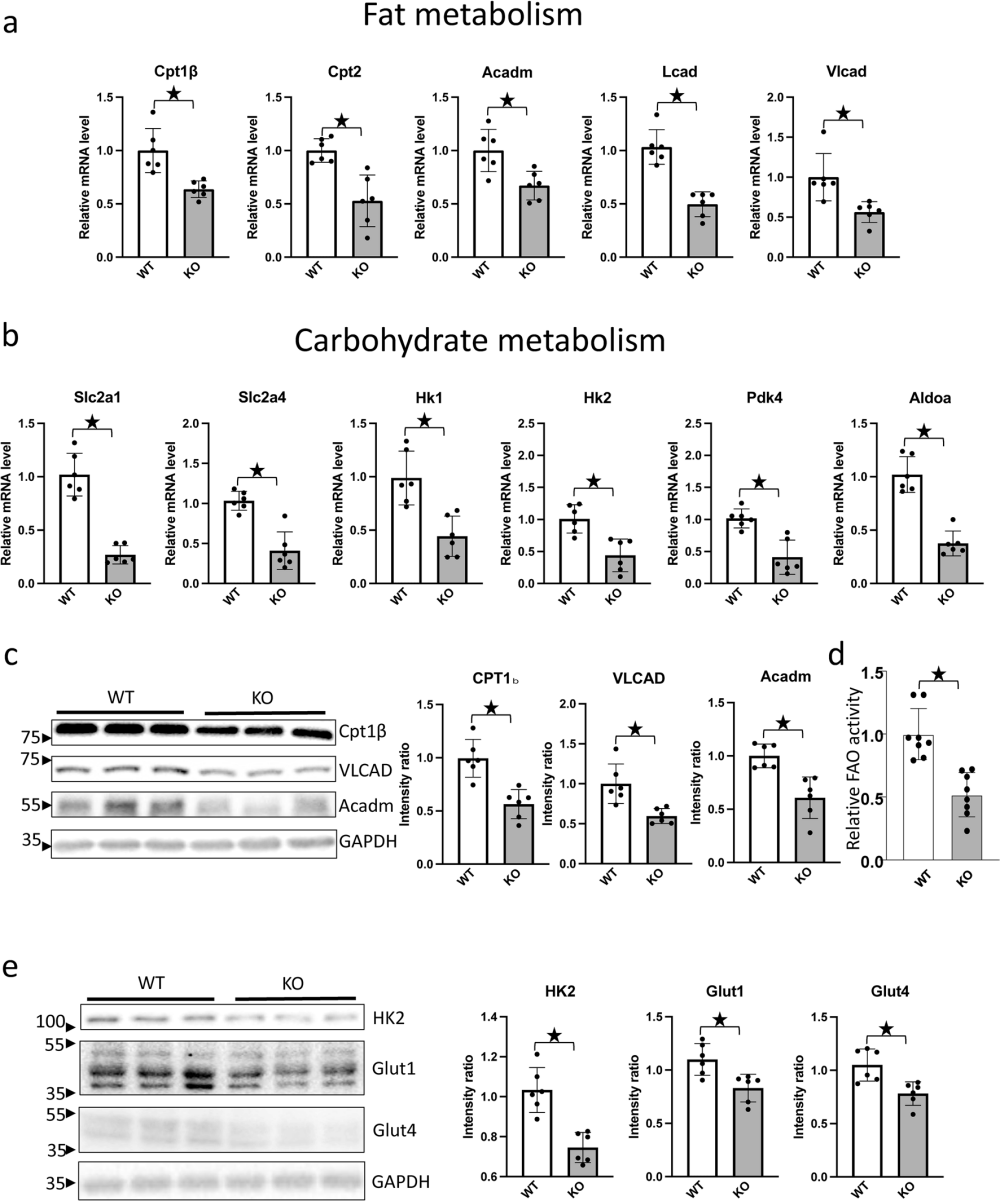
*Perm1* knockout downregulated fat and carbohydrate metabolism-related proteins. (**a**) Expression levels of fat metabolism genes in *Perm1* KO mice. Expression levels of the indicated fat metabolism genes were examined by RT-PCR. Samples were normalized by *15S* gene expression, including *Cpt1β, Cpt2, Mcad, Lcad, and Vlcad*. The mean value from WT mice was expressed as 1 (N = 6), WT (3 male + 3 female); KO (3 male + 3 female), 2 months old. (**b**) Expression levels of carbohydrate metabolism genes in *Perm1* KO mice. Expression levels of the indicated fat metabolism genes were examined by RT-PCR. Samples were normalized by *15S* gene expression, including *Slc2a1, Slc2a4, Hk1, Hk2, Pdk4, and Aldoa*. The mean value from WT mice was expressed as 1 (N = 6), WT (3 male + 3 female); KO (3 male + 3 female), 2 months old. (**c**) Western blots showing CPT1β, VLCAD, and MCAD protein levels. The intensity was normalized by GAPDH (N = 6), WT (3 male + 3 female); KO (3 male + 3 female), 2 months old. (**d**) The heart lysate of WT and *Perm1 KO* mice were analyzed with a fatty acid oxidation (FAO) assay kit. The level of FAO was normalized by that in WT mice. WT (4 male + 4 female); KO (4 male + 4 female), 2 months old. (**e**) Western blots showing HK2, GLUT1, and GLUT4 protein levels. The intensity was normalized by GAPDH (N = 6), WT (3 male + 3 female); KO (3 male + 3 female), 2 months old. The statistical significance was determined with Student’s *t* test, *p < 0.05 was considered significant.

To confirm the results of the RNA sequencing analysis, RT-PCR and Western blot analyses were conducted, using heart homogenates obtained from *Perm1* KO and WT mice. RT-PCR analyses showed that mRNA expression of genes involved in fatty acid metabolism, including *Cpt1*β*, Cpt2, Acadm, Lcad* and *Vlcad*, was significantly lower in *Perm1* KO mice than in WT mice (Fig. 4a). mRNA expression of genes involved in carbohydrate metabolism, including *Slc2a1, Slc2a4, Hk1, Hk2, Pdk4 and Aldoa*, was also significantly lower in *Perm1* KO mice than in WT mice (Fig. 4b). Similar results were obtained at the protein level. The protein levels of CPT1β, VLCAD and Acadm, molecules involved in fatty acid metabolism, were significantly lower in *Perm1* KO mice than in WT mice (Fig. 4c). Cosistently, fatty acid oxidation activity was attenuated in *Perm1* KO mice compared to in WT mice (Fig. 4d). Although the mRNA expression of many genes involved in fatty acid metabolism is downregulated in *Perm1* KO mice, some, including *Hadha*, are upregulated (Supplementary Table S2). HADHA catalyzes the last three steps of β-oxidation of long chain fatty acids. The contribution of the upregulated genes to overall fatty acid oxidation remains to be elucidated. The upregulation of *Hadha* may be compensatory for the genes involved in fatty acid oxidation that are downregulated. Alternatively, downregulation of PERM1 may directly increase transcription of *Hadha* through an unknown mechanism.

The protein levels of HK2, GLUT1, and GLUT4, molecules involved in carbohydrate metabolism, were also lower in *Perm1* KO mice than in WT mice (Fig. 4e).

### PERM1 promotes PPARα-mediated transcription

**Figure 5:**
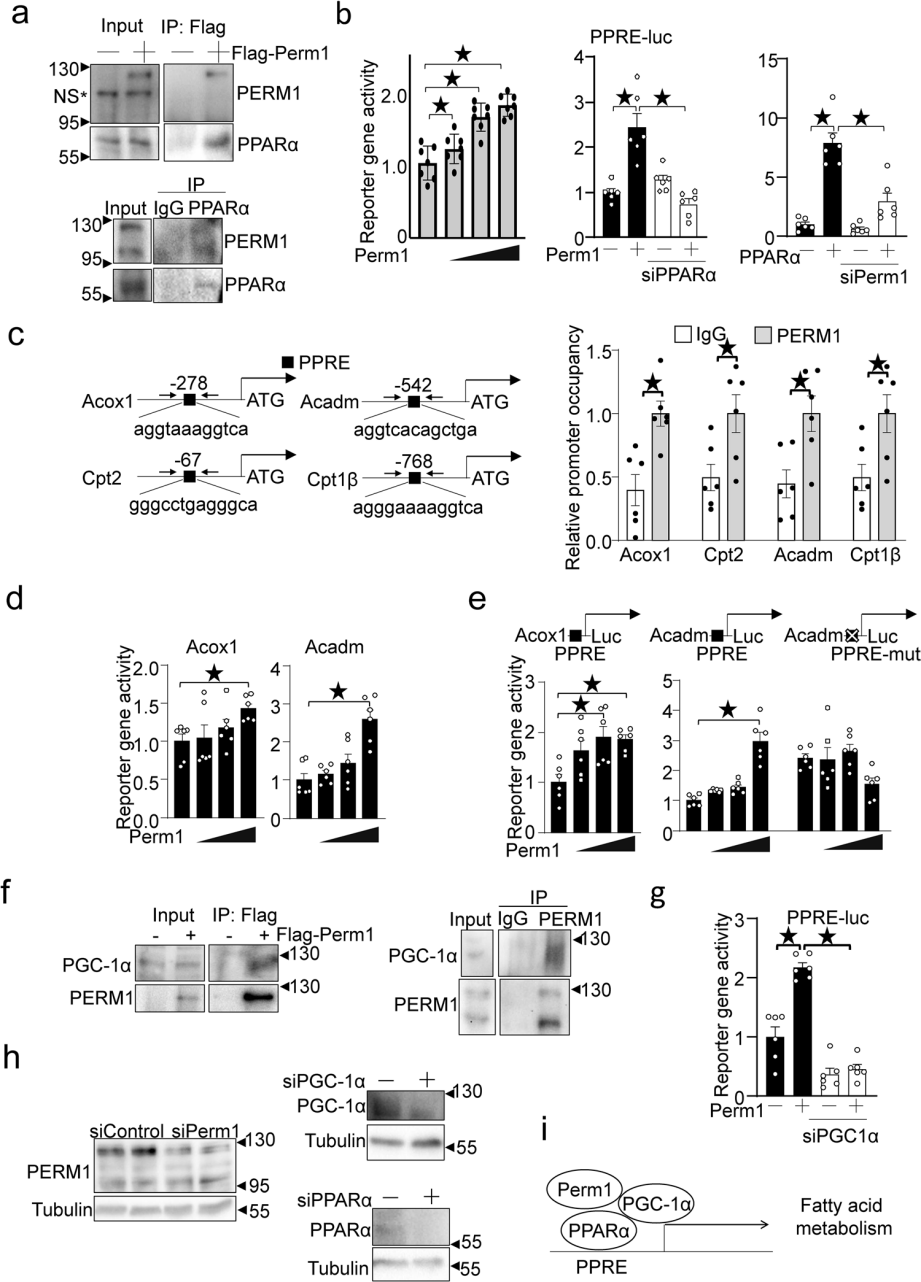
PERM1 promotes PPARα/PGC-1α-mediated transcription. (**a**) (Upper panels) Flag-PERM1 adenovirus was transduced in neonatal rat ventricular cardiomyocytes. Immunoprecipitation was conducted with anti-Flag antibody. Note that endogensou PERM1 in inputs is not well seen due to a short exposure time to avoid overexposures of exogenous PERM1. NS* indicates a non-specific band around 110 kD. (Lower panels) Co-immunoprecipiatation assays were performed using mouse heart lysagte with anti-PPARα antibody and control IgG. Immunoblot analyses were conducted with anti-PERM1 or PPARα antibody. (**b**) PPRE-Luciferase (PPRE-Luc) reporter gene assays were performed with indicated expression vectors and siRNAs. Scrabmled siControl was used as control. N = 6. (**c**) (Left panel) A schematic representation of endogenous PPRE in PPAR target gene promoters including *Acox1, Cpt2, Acadm,* and *Cpt1β*. Arrows indicate primers used for ChIP assays. (Right panel) ChIP assays were were conducted using mouse heart tissue with anti-PERM1 antibody and control IgG. N = 6. (**d**) A luciferase reporter gene horboring the endogenous promoter sequence of either Acox1 andAdcam was co-transfected with increasing concentrations of mammalin expression vector harbaring PERM1 in cultured neonatal rat ventricular myocytes. N = 6. (**e**) A luciferase reporter gene horboring the intact or mutated PPRE sequences on the endogenous Acox1 or Acadm was co-transfected with increasing concentrations of mammalin expression vector harbaring PERM1 in cultured neonatal rat ventricular myocytes. N = 6. (**f**) (Left panel) Representative Western blot image demonstrating that PERM1 interacts with PGC-1α in rat ventricular cardiomyocytes. Cells were transduced with Flag-PERM1 adenovirus for 1 day. Immunoprecipitation (IP) with Flag antibody and immunoblot with PGC-1α antibody. (Right panel) A representative Western blot image demonstrating that endogenous PERM1 interacts with PGC-1α in the heart. Co-immunoprecipiatation assay was performed using mouse heart lysate with anti-PERM1 antibody and control IgG. Immunoblot analyses were conducted with anti-PERM1 or PGC-1α antibody. (**g**) Luciferase reporter assays demonstrating that PERM1-induced reporter gene activation via PPRE was inhibited by knockdown of PGC-1α with siPGC-1α. N = 6. (**h**) The effect of siRNA used by this study including siPerm1, siPPARα and siPGC-1αin neonatal rat ventricular cardiomyocytes. Experiments are repeated 3 times. (**i**) Scheme demonstrating how PERM1 regulates PPARα/PGC-1α-mediated transcription of fatty acid oxidation genes. The statistical significance was determined with 1 way ANOVA (**b** (left), **d**,**e**), Student’s *t* test (**c**) and 2 way ANOVA (**b** (middle and right) and **g**). *p < 0.05 was considered significant (**b**–**e**,**g**).

Since the IPA transcription factor analysis suggested that PERM1 affects the activity of PPARα, a nuclear receptor encoded by *Ppara* and involved in the transcription of the fatty acid metabolism genes, with the highest significance, we further investigated how PERM1 affects PPARα function. We first investigated whether PERM1 interacts with PPARα. Flag-PERM1 was expressed with adenovirus transduction in cultured cardiomyocytes and immunoprecipitaed with anti-Flag antibody. PPARα was co-immunoprecipitated with Flag-PERM1 only when Flag-PERM1 was expressed (Fig. 5a, upper panel). In this experiment, overexpression of Flag-PERM1 by full length cDNA produced only a 120kD protein. To test whether PERM1 interacts with PPARα in the heart, endogenous PPARα was immunoprecipitated from the mouse heart. PERM1 was detected in the anti-PPARα immunoprecipitates but not in control immunoprecipitates (Fig. 5a, lower panel). The activity of a luciferase reporter gene containing three repeats of the consensus PPAR response elements (PPREs) was dose-dependently increased by upregulation of PERM1 in cultured cardiomyocytes (Fig. 5b, left). PERM1-induced increases in PPRE-luciferase activity were inhibited by PPARα downregulation (Fig. 5b, middle). PPARα-induced increases in PPRE-lucsiferase activity were also inhibited by PERM1 downregulation (Fig. 5b, right). These results suggest that endogenous PERM1 plays an important role in stimulating PPARα-induced transcription in a cell-autonomous manner. ChIP assays using mouse hearts showed that PERM1 interacts with the proximal region of PPREs located in the promoters of genes involved in fatty acid metabolism, including *Acox1*, *Cpt2*, *Acadm* and *Cpt1β*, the known targets PPARα (Fig. 5c). To test whether PERM1 stimulates transcription via the endogenous promoter sequences, we constructed reporter genes harboring the endoghenous promoter sequence of the *Acox1* and *Acadm* genes. The activity of the luciferase reporter gene was increased dose-dependently by PERM1 (Fig. 5d). To futher test whether PERM1 stimulates transcription via endogenous PPRE sequences, endogenous PPRE in the *Acox1* or *Acadm* promoters was cloned into the luciferase reporter gene with a minimal promoter. As shown in Fig. 5e, PERM1 stimulated reporter gene activies driven by the endogenous PPRE either on the *Acox1* or *Acadm* promoter, but not PPRE mutant on the *Acadm* promoter, suggesting tht PERM1 stimulates transcription via PPRE-sequence depedenet manner.

### PGC-1α plays an essential role in mediating PERM1-induced activation of PPARs

Previous studies have suggested that PERM1 functions together with PGC-1α, a transcription co-factor that interacts with PPARα and ERRα^8^ Transcription factor analysis also revealed that PGC-1α encoded by *Ppargc1a*, might also be involved in the gene expression profile in *Perm1* KO mice (Fig. 3c). We evaluated whether PGC-1α and PERM1 interact with one another. To this end, Flag-PERM1 was expressed with an adenovirus vector in cultured cardiomyocytes, and immunoprecipitated with anti-Flag antibody. PGC-1α was co-immunoprecipitated with Flag-PERM1 (Fig. 5f, left panel). To test whether PERM1 interacts with PGC-1α in the heart, endogenous PERM1 was immunoprecipitated from the mouse heart. PGC-1α was detected in the anti-PERM1 immunoprecipitates but not in control immunoprecipitates (Fig. 5f, right panel). These results suggest that PERM1 and PGC-1α physically interact with one another. PERM1-induced transcriptional activation via PPRE was abolished in the presence of PGC-1α downregulation (Fig. 5g). The effect of siRNAs was verified with western blot analyses (Fig. 5h). Taken together, these results suggest that PERM1 stimulates transcription via PPREs through a PGC-1α-dependent mechanism in a cell-autonomous manner. Thus, PERM1 may interacts with both PGC-1α and PPARα. In summary, our results suggest that endogenous PERM1 plays an essential role in mediating PGC1α-PPARα-mediated transcription of genes involved in fatty acid metabolism in the mouse heart in vivo at baseline (Fig. 5i). However, scince the evaluation of the ternary complex is based on co-immunoprecipitation and reporter assays with an artificial promoter fragment, further investigation is required to provide unequivocal evidence that the nuclear complex consisting of Perm1, PGC-1α and PPARα mediates fatty acid oxidfation gene expression in vivo.

## Discussion

Previous investigations primarily conducted with in vitro cell cultures have shown that PERM1 is expressed in skeletal and cardiac muscles and involved in the expression of genes regulating metabolism^1,2^. However, the role of endogenous PERM1 in animals in vivo has not been fully understood. Unbiased bioinformatic analyses of the *Perm1* KO hearts and validation with biochemical assays suggest that endogenous PERM1 in the heart mediates expression of genes involved in metabolism in part through a PPARα and PGC-1α dependent mechanism in the heart.

*Perm1* KO mice were born in the expected Mendelian ratio and their cardiac phenotype was apparently normal at young ages. PERM1 appears to be dispensable during fetal development and postnatal growth of the heart in both male and female mice. RNA sequencing analysis and bioinformatic analyses showed, however, that PERM1 critically regulates genes involved in both fatty acid and carbohydrate metabolism in the heart at baseline consistent with a recent report. Genes involved in fatty acid metabolism, including *Lcad, Acadm, Vlcad, Cpt1β, Cpt2, Acox1,* and *Acox2,* are downregulated in *Perm1* KO mouse hearts as are some genes involved in carbohydrate metabolism, including *Slc2a1, Slc2a4, Pdk,* and *Hk.* Our results are consistent with an in vitro study conducted using cultured cardiomyocytes, in which downregulation of endogenous PERM1 caused suppression of metabolic genes involved in the TCA cycle, fatty acid oxidation, and glycolysis^2^. Fatty acid oxidation evaluated with heart homogenates was significantly attenuated in *Perm1* KO mice compared to in WT mice. Genetic depletion of *Perm1* partially mimics the downregulation of genes involved in fatty acid oxidation during heart failure. Despite the downregulation of key genes involved in fatty acid metabolism, however, the cardiac function of young *Perm1* KO mice was normal at baseline, suggesting that downregulation of PERM1 alone is not sufficient to trigger cardiac dysfunction in the mouse heart at 3 months of age. The function of the heart is compensated at baseline despite downregulation of metabolic genes, most likely through activation of neurohormonal effects. A similar phenotype has been reported in young PGC-1α KO mice^11^. Since PGC-1α KO mice gradually develop mild cardiac dysfunction at 7–8 months, it is possible that *Perm1* KO mice may also exhibit cardiac dysfunction later in their life or in response to stress.

A recent proteomic study conducted with systemic *Perm1* KO mice exhibited downregulation of mitochondrial proteins, including LIPIN1, solute carrier 25 (SLC25) family proteins, and CPT2^9^. The changes in the levels of some mitochondrial proteins were accompanied by increases in the levels of phosphatidic acid, phosphatidylinositol and phosphatidylethanolamine, and basic and branched chain amino acids, and decreases in the level of acylcarnitines, without significant alteration in the levels of ATP, ADP, pyruvate, lactate, and TCA cycle intermediates. Whether downregulation of mitochondrial transporter proteins occurs through transcriptional mechanisms remains to be elucidated.

RNA sequencing and transcription factor binding site analyses suggested that downregulation of PERM1 affect gene expression through mechanisms dependent upon PPARα and PGC-1 (Fig. 3c). The analysis also suggested downregulation of genes regulated by other transcription factors, including PPARγ, KLF15 and ERRα, which share target genes with PPARα^12,13^. PPARα belongs to the PPAR family of ligand-activated nuclear receptors, consisting of three members: alpha, beta/delta, and gamma. Among these receptors, PPARα regulates lipid metabolism^14–18^. We found that PERM1 physically interacts with PPARα in the heart. ChIP-qPCR assays indicated that PERM1 interacts with the promoters of genes involved in fatty acid metabolism and regulated by PPARα. Furthermore, downregulation of PERM1 inhibited PPARα-mediated transcription in cultured cardiomyocytes in vitro. Taken together, these results are consistent with the notion that PERM1 interacts with PPARα and positively regulates transcription of PPARα target genes involved in fatty acid metabolism in the heart. The involvement of other transcription factors, including ERRα, in mediating the effect of PERM1 in vivo remains to be investigated.

How does PERM1 affect the transcriptional activity of PPARα? Currently, the molecular identity of PERM1 remains unclear. It has been reported that PERM1 is located in both the nucleus and the cytosol^1^. In our previous study, we also showed that PERM1 is translocated to the nucleus in response to phenylephrine, a hypertrophic stimulus^2^. Hence, we hypothesize that PERM1 can interact with PGC1α and PPARα in the nucleus. We here show that PERM1 interacts with PPARα. It has been shown that PGC-1α, a transcription coactivator, interacts with PPARα and stabilizes PPARα binding to the promoters of target genes^19^. We here also show that PERM1 interacts with PGC-1α and that downregulation of PGC-1α inhibits PERM1-induced stimulation of the PPRE-luciferase reporter. These results suggest that PERM1 stimulates the transcriptional activity of PPARα in a PGC-1α-dependent manner. One possibility is that PERM1 serves as a scaffold, thereby stabilizing PGC-1α-PPARα interaction to upregulate PPARα target genes. A recent study suggested that PERM1 located on the outer mitochondrial membrane affects the levels of phospholipid species^9^. Whether PERM1 locally affects the level of mitochondrial proteins through transcription-independent mechanisms in the heart remains to be elucidated.

Our results suggest that mRNA expression of genes involved in carbohydrate metabolism is also downregulated in *Perm1* KO mice. The underlying mechanism through which PERM1 regulates carbohydrate metabolism is currently unknown. It has recently been shown that PERM1 is protective against ischemia in cultured cardiomyocytes in vitro^8^. Since ischemia stabilizes HIF-1α signaling to upregulate glycolysis^20–22^, PERM1 may promote expression of genes involved in carbohydrate metabolism through activation of the HIF-1α pathway. Further investigation is needed to test this hypothesis.

We used the CRISPR-Cas9 system to generate systemic KO mice. It is possible that genome editing induced a non-specific or off-target alteration in the genome besides the deletion of the DNA segment in exon 2 of the *Perm1* gene^23–26^. To minimize this potential problem, we established two lines of KO mice, namely line 29 and line 80, with deletions of 1157 bp and 1346 bp in exon 2, respectively. We confirmed that both lines exhibit complete deletion of PERM1. Both lines showed a similar baseline phenotype after breeding through multiple generations. Thus, it is likely that the deletion of the *Perm1* gene specifically causes the phenotype reported here.

In conclusion, endogenous PERM1 interacts with PPARα and PGC-1α and regulates genes involved in fatty acid in the heart at baseline. These mice should be useful for investigating the role of endogenous PERM1 in vivo at baseline, including during aging, and in response to stress.

## Methods

All methods were carried out in accordance with relevant guidelines and regulations.

### Generation of *Perm1* homozygous knockout (KO) mice

*Perm1* systemic homozygous KO mice (C57BL/6 background) were generated in the Genome Editing Shared Resource of Rutgers-Cancer Institute of New Jersey. Briefly, mouse *Perm1* KO was made by coinjecting two CRISPR gRNAs (MilliporeSigma) with Cas9 protein (MilliporeSigma) into C57BL/6J zygotes to generate a deletion in the coding sequence (Online Fig. 1). The gRNAs targeted the sequences 5′-TGATGAGTGTGGCCTCTTGCAGG-3′ and 5′-GAAGCTGCCATGGTGGATACAGG-3′ (PAM underlined). Founder mice were genotyped to detect the approximate 1.3 kb deletion. Primers *Perm1*A 5′-ATCCTGGATTTGTGAAGACCCTGC-3′ and *Perm1*D 5′-ACGGCTTCCTGCTGCTGTCCTG-3′ were used to amplify the alleles. Wild-type alleles in the founders were 1716 bp, whereas deletion alleles were approximately 384 bp, depending on the precise deletion. Founder 80, which was used for subsequent experiments, had a deletion of 1346 bp, which encompassed almost 2/3 of the coding sequence of exon 2. All experiments were performed with randomization and allocation concealment and in a blind fashion. All experiments involving animals were approved by the New Jersey Medical School Institutional Animal Care and Use Committee. All methods are reported in accordance with ARRIVE guidelines (https://arriveguidelines.org) for the reporting of animal experiments.

### Genotyping

The toes were cut from mice at about 4 weeks of age. The tissue DNA was extracted using tissue extraction buffer (E7526, Extraction solution and T3070, Tissue Preparation solution, Sigma). The sample was incubated for 15–20 min at 55 °C and then at 95 °C for 3 min. After that, the sample was neutralized (N3910, Neutralization solution B) and the DNA solution was added to PCR premix solution (RR320B, EmeralAm@MaxPCR MasterMIx, Takara). The primer sequences used were:

*Perm1* WT Fw:TCTGCCTCGTTCTGTTTCAAAGACTG

*Perm1* #29 KO Fw:AGCAGCTGGTCAACAGGTGTGT

*Perm1* #80 KO Fw:TGATGAGTGTGGCTGTACCTCCA

Perm1 Rv:CACAGCATCCCAGGCTGCAGA

PCR was carried out using a protocol of 30 cycles of 94 °C for 30 s in the denaturing stage, 60 °C for 30 s in the annealing stage, and 72 °C for 30 s in the extending stage. The PCR product was checked on a TAE gel. Products with one band of 361 bp indicate wild type, with one band of 270 bp indicate #29 *Perm1* homozygous KO, with one band of 260 bp indicate #80 *Perm1* homozygous KO, and with two bands indicate heterozygous KO.

### Immunoblot analyses

Heart homogenates were prepared in RIPA lysis buffer containing 50 mmol/L Tris (pH 7.5), 150 mmol/L NaCl, 1% IGEPAL CA-630 (Sigma Aldrich), 0.1% SDS, 0.5% deoxycholic acid, 10 mmol/L Na_4_P_2_O_7_, 5 mmol/L EDTA, 1:100 diluted Protease Inhibitor Cocktail (Sigma Aldrich, P8340-5ML), and 1:100 diluted Phosphatase Inhibitor Cocktail (Sigma Aldrich, P0044-5ML). Protein amounts were measured by BCA quantification and 20–30 μg were subjected to 10–15% SDS-PAGE. After proteins were transferred to a 0.2 μm PVDF membrane, immunoblots were probed with the indicated antibodies.

### Antibodies

The following primary antibodies were used for immunoblots: Anti-Hexokinase II (Cell Signaling Technology, 2867), Anti-GLUT1 (Cell Signaling Technology, 12939S), Anti-GLUT4 (Cell Signaling Technology, 2213S), Anti-CPT1β (Abcam, ab134988), Anti-VLCAD (Abcam, ab155138), Anti-Acadm (Santa Cruz, sc365108), Anti-PPARα (Cayman, 101710 and Novus Biologicals, N300-537), Anti-PGC-1α (Millipore, Ab3242), Anti-GAPDH (Cell Signaling Technology, 2118C), Anti-Tubulin (Cell Signaling Technology, 3873S). Secondary antibodies used were Anti-Rabbit IgG, HRP-link (Cell Signal Technology, 7074S) and Anti-Mouse IgG, HRP-link (Cell Signal Technology, 7076S). In all Western blot images shown in figures, individual lanes correspond to individual mice. Molecular weights (MW) are shown. The signal intensity of Western blots was quantified using ImageJ software and then normalized by the corresponding loading control (Tubulin or GAPDH).

### Echocardiography

Mice were anesthetized using 12 µ/g body weight of 2.5% Avertin (Sigma-Aldrich). Echocardiography was performed by ultrasonography (Acuson Sequoia C256; Siemens Medical Solutions) using a 13-MHz linear transducer. After chest shaving, the mouse was fixed on the platform and needle electrodes were attached for electrocardiography. B-mode and M-mode tracings (seep speed = 100-200 mm/s) were recorded from the parasternal short-axis view at the mid-papillary muscle level.

### Histological analyses

The LV accompanied by the septum was cut into base, middle portion, and apex, fixed with 10% formalin, embedded in paraffin, and sectioned at 4–6 μm thickness. The sections were incubated in 3% H_2_O_2_ in PBS to prevent endogenous peroxidation and blocked with 5% BSA in PBS. Cardiomyocyte cross-sectional area was measured from images captured of sections stained with wheat germ agglutinin (WGA). Cardiomyocyte size was measured using ImageJ software. The mean size of cardiomyocytes in WT mice was used as a reference. Interstitial fibrosis was measured using Picro Sirius Red (PSR) staining and ImageJ software. The total percentage of the area of fibrosis was calculated as the sum of red-stained areas divided by total ventricular area. The mean fibrotic area in WT mice was used as a reference.

### RNA-seq

Total RNA was extracted from the heart tissue of WT and *Perm1* KO mice by the TRIzol protocol. WT mice were used as a control group. Samples were eluted in RNase/DNase-free water. Sample quality control, library preparation, sequencing and alignments were performed by the LC Sciences company and Cancer Progression Research Center, National Yang-Ming Chiao-Tung University. The differential expression sequence (DES) gene result was selected based on an adjusted P value less than 0.05. These genes were analyzed using the DAVID Bioinformatics Resource 6.8. According to gene function and pathway annotation, KEGG and GO graphs were designed using GraphPad Prism 8 (GraphPad Software, LLC). The expression levels of indicated fat and carbohydrate metabolism genes in *Perm1* KO mice were compared with those in WT mice. The transcription factor analysis was performed by Ingenuity Pathway Analysis (IPA, Qiagen, Hilden, Germany). The activation z-score and p-value of overlap indicate the presence of a statistically significant overlap between dataset genes and known targets regulated by a given regulator.

### Real-time quantitative polymerase chain reaction (qRT-PCR)

qRT-PCR was performed using self-designed primers ordered from the IDT company. Tissue RNA was extracted using TRIzol (Life Technologies, 15,596,018), followed by isopropanol. One µg mRNA was used for reverse transcription using the TB Green Premix Ex Taq II system according to the manufacturer’s instructions (TaKaRa, RR820B). The Cq and starting quantity values from the targeted genes were normalized by the gene expression level of 15S. The ratio of each sample was compared with the average ratio of WT mice.

### Immunoprecipitation

For in vitro experiments, neonatal ventricular cardiomyocytes were plated in 10 cm dishes. Cells were transduced with adenovirus harboring Flag-PERM1. The cells were harvested after one day. Immunoprecipitation was carried out by incubating with anti-Flag M2 affinity gel (Sigma Aldrich, A2220) overnight at 4 °C. The beads were washed and 2 × sample buffer was added to produce the final sample. For in vivo experiments, hearts were harvested and homogenized in lysis buffer. For immunopreciption wth anti-PERM1 antibody, the lysate was incubated by anti-IgG (Cell Signaling Technology, 2729) or anti-PERM1 antibody (Sigma Aldrich, HPA031711) overnight at 4 °C. For immunopreciption with PPARα, the lysate was incubated with anti-IgG (Cell Signaling Technology, 5415) or anti-PPARα antibody (Novus Biologicals, N300-537) overnight at 4 °C. Protein A/G-Agarose (Santa Cruz, sc2003) was added the next day for one hour. The final steps were the same as for in vitro immunoprecipitation. Immunoblot was conducted by primary anti-PPARα (Cayman, 101719), anti-PGC1α (Abcam, ab54481) or anti-PERM1 antibody.

### siRNAs

Small interfering RNA (siRNA) for rat *Perm1* (SI02021775), rat PGC-1α (SI00271012), and rat PPARα (SI01963451) and control siRNA (1022076) were obtained from Qiagen. The siRNA was transfected using Lipofectamine RNAiMax (Invitrogen). Four and a half µl of Lipofectamine RNAiMax was diluted with 125 µL OPTI-MEM and 2 µL of 20 µmol/L siRNA was diluted with 125 µL OPTI-MEM. The diluted Lipofectamine RNAiMax and siRNA were then mixed and incubated at room temperature for 20 min. After incubation, the mixture was added to cardiomyocytes plated on a 3.5 cm dish with 2 mL OPTI-MEM.

### Adenovirus vector

The *Perm1* sequences were cloned from mouse cDNA. pDC316 plasmid was digested by restriction enzymes. The *Perm1*-Flag sequence was then cloned into the digested pDC316 plasmid (pDC316-Perm1-Flag). After sequencing, HEK293 cells were used for adenovirus packaging and amplification. Adenovirus harboring β-galactosidase (Ad-LacZ) was used as a control.

### Luciferase reporter assays

Luciferase reporter assays were conducted using a luciferase assay system (Promega, E4550). Neonatal ventricular cardiomyocytes were plated in a 12-well dish. Reporter plasmids (0.3 µg per well) including 3xPPRE-luc, pGL3-Acox1 promoter, pGL3-Acadm promoter, Acox1-PPRE-luc, Acadm-PPRE-luc and Acadm-PPREmutant-luc was transfected together with PERM1 expression vector (pDC316-Perm1, 0.03, 0.07 and 0.1 µg) using Lipofectamine 2000 (Invitrogen, 11668-019) for 24 h. For knockdown experiments, reporter plasmd (0.3 µg per well), mammalian expression vector such as PPARα (pDC36-PPARα) or Perm1 (0.3 µg per well), and siRNA (40 pMol) such as siScrable, siPPARα, siPGC-1α and siPerm1, were transfected with Lipofectamine 2000. pDC316 control vector was used so that total plasmids were equal at 1 µg per well. The cells were harvested in 100 µL Reporter lysis buffer (Promega, E3971) per well after 2–3 days of transfection. Luminescence was normalized by protein content. The average value of the control group (pure pDC316 vector and siScramble) was expressed as a relative value of 1.0. Reporter plasmids, including PPRE-luciferase, were described previously^2^.

### Reporter plasmids

Mouse endogenous 5′ promoter region of Acox1 (505 bp) and Acadm (700 bp) from start codon were cloned into pGL3 basic vector to generate pGL3-Acox1 promoter and pGL3-Acadm promoter. Endognous PPRE sequence in Acox1 promoter (AACGTGACCTTTGTCCTGGTC), Acadm (GTAAAGGTCACAGCTGACTGC) and its mutant (GTAAAGGTCACAAATGACTGC) were cloned into pGL3basic with 32 bp of minimal promoter (TAGAGGGTATATAATGGAAGCTCGACTTCCAG) to generate Acox1-PPRE-luc, Acadm-PPRE-luc and Acadm-PPREmutant-luc.

### Chromatin-immunoprecipitation (ChIP)-PCR

Heart tissue from 2-month-old WT mice was cross-linked using formaldehyde. The chromatin was extracted from the nuclear fraction by sonication and immunoprecipitated with anti-PERM1 antibody (Sigma Aldrich, HPA031711). RT-PCR of the chromatin fragment was conducted using Maxima SYBR Green qPCR master mix (ThermoFisher, K0223). The primers were designed as: *Acox1*-*GGAAAGATCACGTGAACCTGGAG* and *TCCTCACGTGACCGGCTGCAAT*; *Cpt2*-*CCCTGTGGGCGGAGTTGAACT* and *GCGGTGGCTGAGGGAGTTCC*; *Acadm*-*CTCTCCAAGTAAAGGTCACAGCTG* and *ACTGTGTGCCCAGTGTCACCG*; *Cpt1β*-*AACCTTGAGCCCTGGAATTAGGGA* and *AGGGTCTCACGTGAGCATGGT*.

### Fatty acid oxidation (FAO) activity

FAO activity was analyzed using Fatty Acid Oxiation Assay Kit (E-141, Biomedical Research Service Center, University at Buffalo, Atate University of New York). Briefly, heart tissues were subjected to homogenization with ice-cold Cell Lysis Solution. The sample was then centrifuged by 14,000 rpm for 5 min, the supernatant was harvested and the protein concentration was determined with the BCA protein assay. The sample was mixed with FAO substrates diluted with the FAO assay solution and incubated 37 °C for 30–60 min. The absorbance at 492 nm was measured.

### Statistics

All values in graphs are expressed as the mean $\pm$ S.E. Normality was tested with the Shapiro–Wilk normality test. If the data exhibited a normal distribution, pairwise testing was performed with the Student’s *t* test and multiple group comparisons were performed by 1-way or 2-way ANOVA, followed by Tukey post-test. A priori power calculations were performed based on data from published studies^27,28^ and pilot experiments. The effect size in this study was 1–5 with alpha = 0.05 and power = 0.80. Microsoft Excel 2016 was used for Student’s *t* tests and GraphPad Prism 9 was used for ANOVA and Tukey post-test. Dot plot was used. All quantified Western blot and cell viability data are shown as values relative to control. The survival rate was calculated by the Kaplan–Meier Curve method.

## Supplementary Information

Supplementary Information 1.

Supplementary Information 2.

Supplementary Information 3.

## Data Availability

The data that support the findings of this study are available from the corresponding author upon reasonable request.
