# Supplementary material for: PERM1 regulates genes involved in fatty acid metabolism in the heart by interacting with PPARα and PGC-1α
Source: Sci Rep. 2022 Aug 26;12:14576. doi: 10.1038/s41598-022-18885-3 (PMC9418182; doi:10.1038/s41598-022-18885-3)
Supplement: Supplementary file 1 — Supplementary Information 1. [file 41598_2022_18885_MOESM1_ESM.pdf]

Supplemental Materials for

PERM1 regulates genes involved in fatty acid metabolism in the heart by interacting with PPARα and PGC-1α

Running title: ***PERM1 regulates PPARα in heart tissue***

Chun-yang Huang[1,2,3], Shin-ichi Oka[1], Xiaoyong Xu[1,4], Chian-Feng Chen[5], Chien-Yi Tung[5], Ya-Yuan Chang[5], Youssef Mourad[1], Omair Vehra[1], Andreas Ivessa[1], Ghassan Yehia[6], Peter Romanienko[6], Chiao-Po Hsu[2,3], and Junichi Sadoshima[1*]

Affiliation:
[1]Department of Cell Biology and Molecular Medicine, Rutgers New Jersey Medical School, Newark, NJ 07103.
[2]Division of Cardiovascular Surgery, Department of Surgery, Taipei Veterans General Hospital, Taipei, Taiwan.
[3]Department of Medicine, School of Medicine, National Yang-Ming Chiao-Tung University, Taipei, Taiwan.
[4]Department of Cardiology, Ningbo Medical Center Lihuili Hospital, Ningbo, Zhejiang, China
[5]Cancer Progression Research Center, National Yang-Ming Chiao-Tung University, Taipei, Taiwan.
[6]Genome Editing Core Facility, Rutgers Cancer Institute of New Jersey, New Brunswick, NJ 08901

[*]Correspondence:
Junichi Sadoshima, MD PhD
Department of Cell Biology and Molecular Medicine,
Rutgers New Jersey Medical School
185 South Orange Ave., MSB G609
Newark, NJ 07103
Tel: (973)972-8619
Fax: (973)972-7489
Email: sadoshju@njms.rutgers.edu

Figure S1

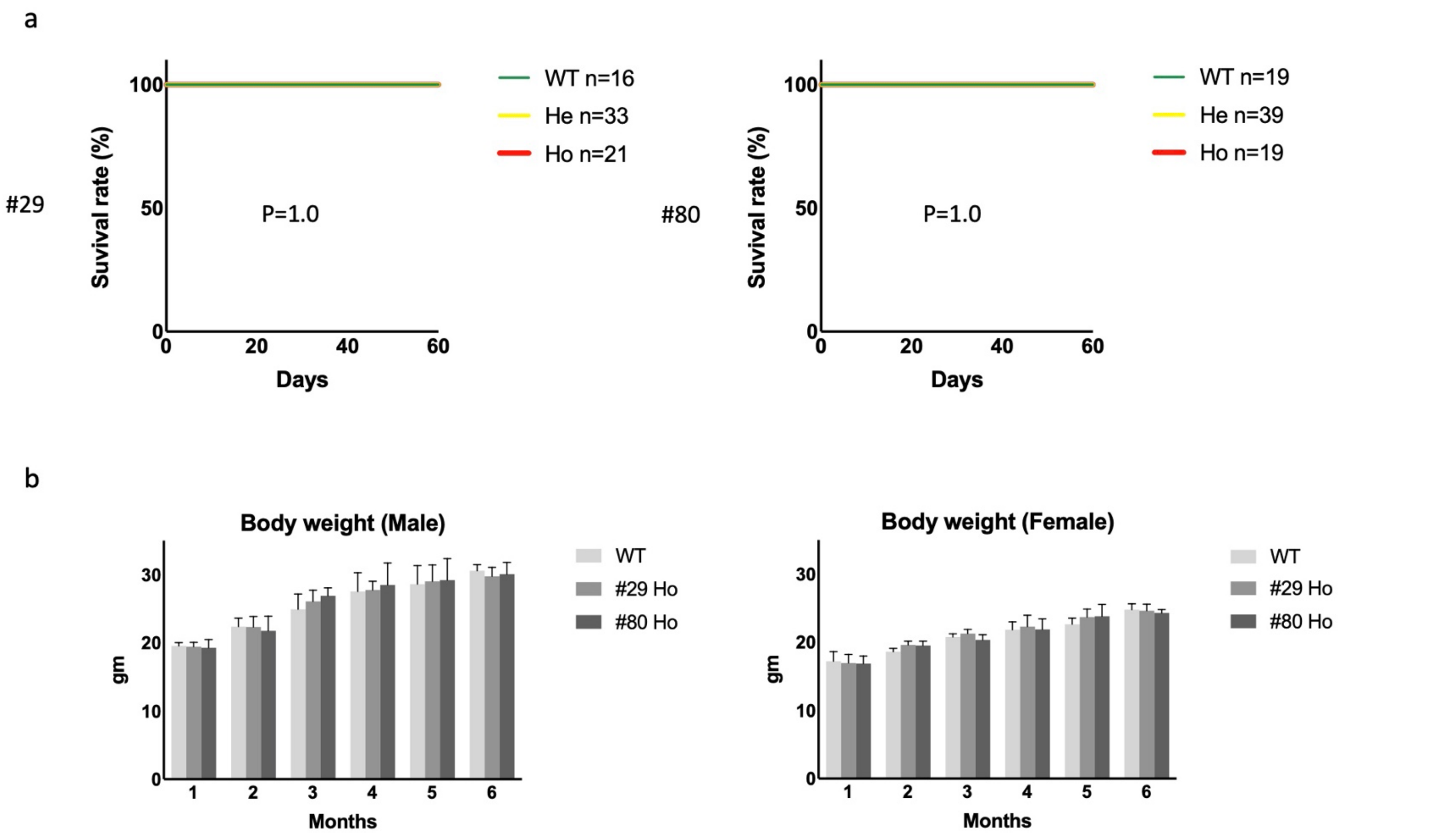

Supplementary Figure 1

a: Left and right panels show two-month survival rate for Line 29 and Line 80.

Line 29: WT (8 male + 8 female); He (16 male + 17 female); Ho (10 male + 11 female)

Line 80: WT (10 male + 9 female); He (20 male + 19 female); Ho (10 male + 19 female)

b: Body weight growth comparisons among WT, Line 29 Ho, and Line 80 Ho for the same gender. Ta

WT (8 male); Line 29 Ho (8 male); Line 80 Ho (8 male); WT (9 female); Line 29 Ho (9 female); Line 80 Ho (9 female)

Table S1

Echocardiographical measurements of WT and *Perm1*KO mice at baseline

Group	*n*	HR (beats/min)	IVSd (mm)	LVIDd (mm)	PWd (mm)	LVIDs (mm)	EF (%)
**WT**	**21**	488±61.6	0.88±0.14	3.49±0.41	1.08±0.36	2.08±0.54	71.2±12.3
**#29 He**	**20**	486±56.9	0.85±0.13	3.23±0.44	1.03±0.26	1.93±0.43	72.1±9.2
**#80 He**	**13**	493±66.9	0.89±0.16	3.24±0.37	1.17±0.43	1.93±0.52	71.2±14.4
**#29 Ho**	**14**	495±48.5	0.87±0.13	3.60±0.35	0.86±0.24	2.10±0.39	68.9±6.6
**#80 Ho**	**20**	511±56.9	0.88±0.16	3.51±0.36	0.98±0.29	2.13±0.4	69.5±11.8

No significant difference, compared with WT

Fat metabolism key genes

Table S2

Gene name	Mean FPKM. WT (n=4)	Mean FPKM. KO (n=4)	P value	Regulation
Acadl	319.50	144.42	0.00	down
Acadm	159.57	39.86	0.00	down
Acads	41.22	15.31	0.04	down
Acadvl	217.94	98.61	0.00	down
Acat1	92.89	109.26	0.01	up
Acat2	1.35	1.25	0.44	
Acat3	0.29	0.20	0.63	
Acox1	35.86	15.38	0.00	down
Acox2	0.11	0.04	0.05	
Acox3	1.60	1.54	0.54	
Acsl1	47.03	50.59	0.10	
Acsl3	1.11	0.47	0.01	down
Acsl4	1.58	1.44	0.13	
Acsl5	4.12	3.80	0.63	
Acsl6	0.71	0.59	0.31	
Acss1	59.04	55.17	0.19	
Acss2	2.31	2.34	0.83	
Acss3	0.10	0.04	0.31	
Cpt1a	4.55	1.49	0.00	down
Cpt1b	119.02	34.94	0.00	down
Cpt1c	0.48	0.23	0.03	down
Cpt2	49.13	23.53	0.00	down
Ech1	340.71	445.91	0.01	up
Echdc1	1.84	2.04	0.46	
Echdc2	10.16	11.11	0.21	
Echdc3	6.88	6.97	0.85	
Eloa	6.01	6.30	0.28	
Elob	38.79	38.96	0.97	
Eloc	10.17	11.94	0.01	up
Fas	4.36	4.77	0.22	
Fasn	1.34	1.09	0.38	
Fat1	1.15	1.00	0.36	
Fat2	0.01	0.01	0.84	
Fat3	0.02	0.02	0.86	
Fat4	0.91	0.76	0.26	
Hacd1	11.95	13.05	0.49	
Hacd2	1.41	1.68	0.08	
Hacd3	5.74	6.20	0.28	
Hacd4	0.99	0.92	0.64	
Hadh	63.80	71.33	0.09	
Hadha	86.97	104.45	0.00	up
Hadhb	98.28	135.82	0.00	up
Slc25a1	2.48	2.57	0.82	
Slc25a2	0.10	0.05	0.40	

Table S3

Carbohydrate metabolism key genes

Gene name	Mean FPKM. WT (n=4)	Mean FPKM. KO (n=4)	P value	Regulation
Aldoa	459.62	475.95	0.67	
Aldob	5.63	1.68	0.20	
Aldoc	0.04	0.01	0.04	down
Eno1	13.43	13.87	0.55	
Eno2	0.18	0.27	0.08	
Eno3	421.27	419.66	0.96	
G6pd2	0.01	0.00	0.39	
Gapdh	49.28	36.33	0.22	
Gpi1	57.59	53.66	0.39	
Hk1	13.52	11.54	0.36	
Hk2	59.30	29.35	0.00	down
Hk3	0.13	0.18	0.17	
Pfkm	167.86	168.49	0.96	
Pgam1	16.53	19.29	0.09	
Pgam2	395.68	439.32	0.25	
Pgam5	7.64	7.66	0.98	
Pgd	2.87	3.19	0.24	
Pkm	121.33	129.20	0.45	
Rpia	1.34	1.95	0.00	up
Slc2a1	2.68	1.21	0.01	down
Slc2a4	88.86	35.42	0.00	down
Taldo1	21.29	21.05	0.90	
Tkt	2.54	2.91	0.39	

RT-PCR primer sequences (5’→3’)

Gene name	Forward	Reverse
Cpt1b	ATGACAGGGGCTGGGATCGAC	GACCAAAGCCACCTCCAGCAC
Cpt2	GGCCAGCTGACCAAAGAAGCAG	GGTGGACAGGATGTTGTGGTTTATC
Mcad	GAAGCTGATGAGGGACGCCA	GCTTGGAGCTTAGTTACACGAGG
Lcad	GGTGGAAAACGGAATGAAAGG	GGCAATCGGACATCTTCAAAG
Vlcad	TATCTCTGCCCAGCGACTTT	TGGGTATGGGAACACCTGAT
Slc2a1	GCTTCTCCAACTGGACCTCAAAC	ACGAGGAGCACCGTGAAGATGA
Slc2a4	CTGCAAAGCGTAGGTACCAA	CCTCCCGCCCTTAGTTG
Hk1	CATTGTCTCCTGCATCTCCGA	ATTCCGCAATCTAGGCTCGTC
Hk2	CTTGCGAATATGGTTGCCTCA	CCTCCCAATGCCTGATCTGAT
Pdk4	AGGTTATGGGACAGACGCTATCATCTACTT	GTTCTTCGGTTCCCTGCTTGGG
Aldoa	GGCCATCATGGAAAATGCC	TCAAGTCATGGTCCCCATCAG

Lysate buffer formula

NaCl	150mM
Tris HCl	50mM, pH 7.4
Triton	1%
ETDA	1mM

Sample buffer formula (5x)

Tris HCl	60mM, pH 6.8
Glycerol	25%
SDS	2%
2-mercaptoethanol	14.4mM
Bromophenol blue	0.1%
