# Supplementary figures and images for: PERM1 regulates genes involved in fatty acid metabolism in the heart by interacting with PPARα and PGC-1α
Source: Sci Rep. 2022 Aug 26;12:14576. doi: 10.1038/s41598-022-18885-3 (PMC9418182; doi:10.1038/s41598-022-18885-3)

Fig. 1C

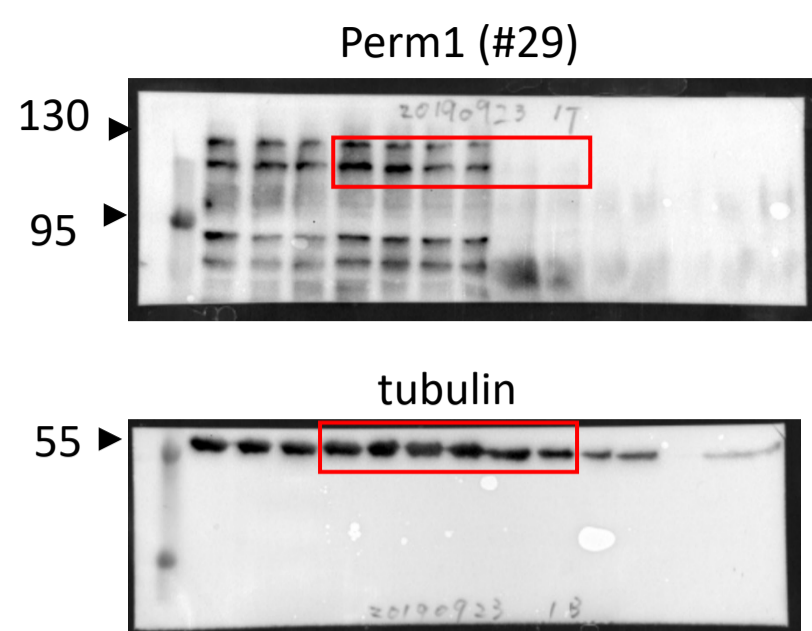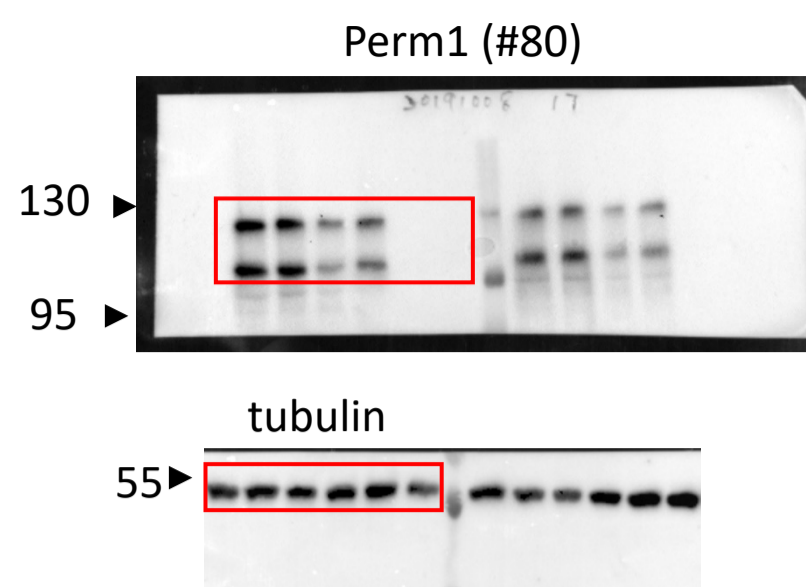

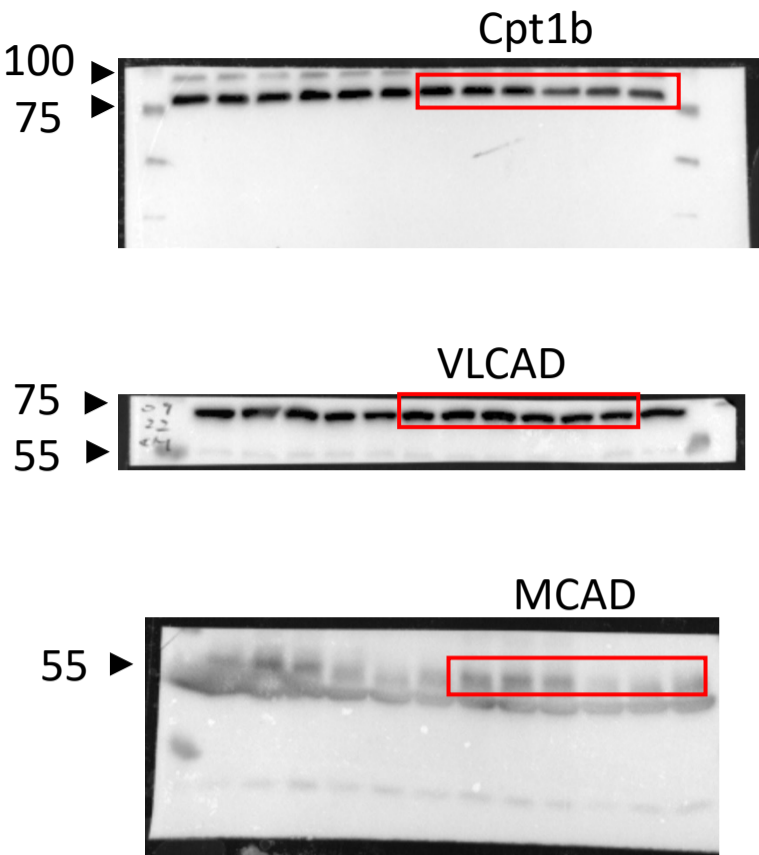

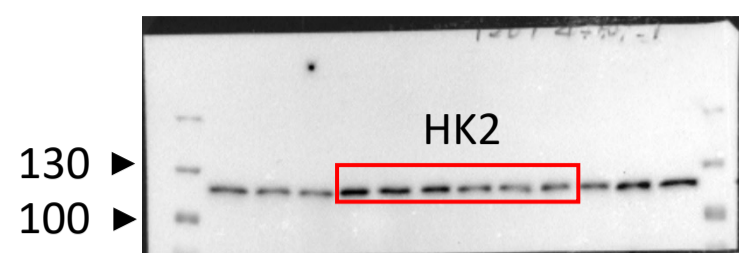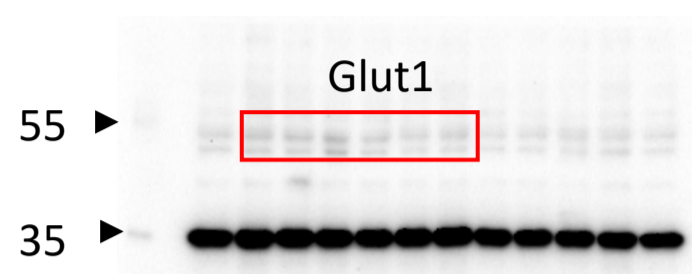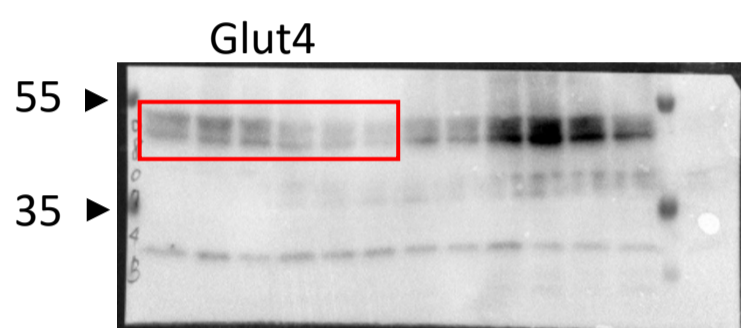

Fig. 5A

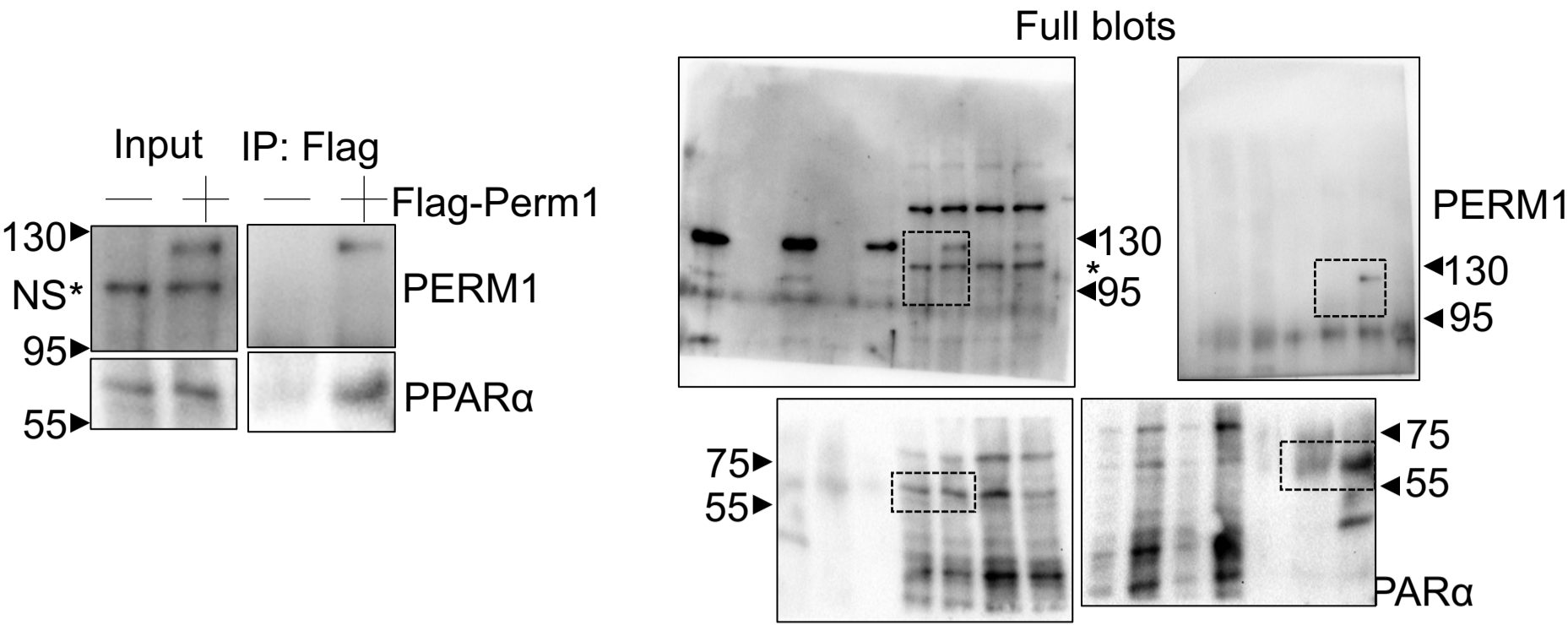

Multiple exposure images

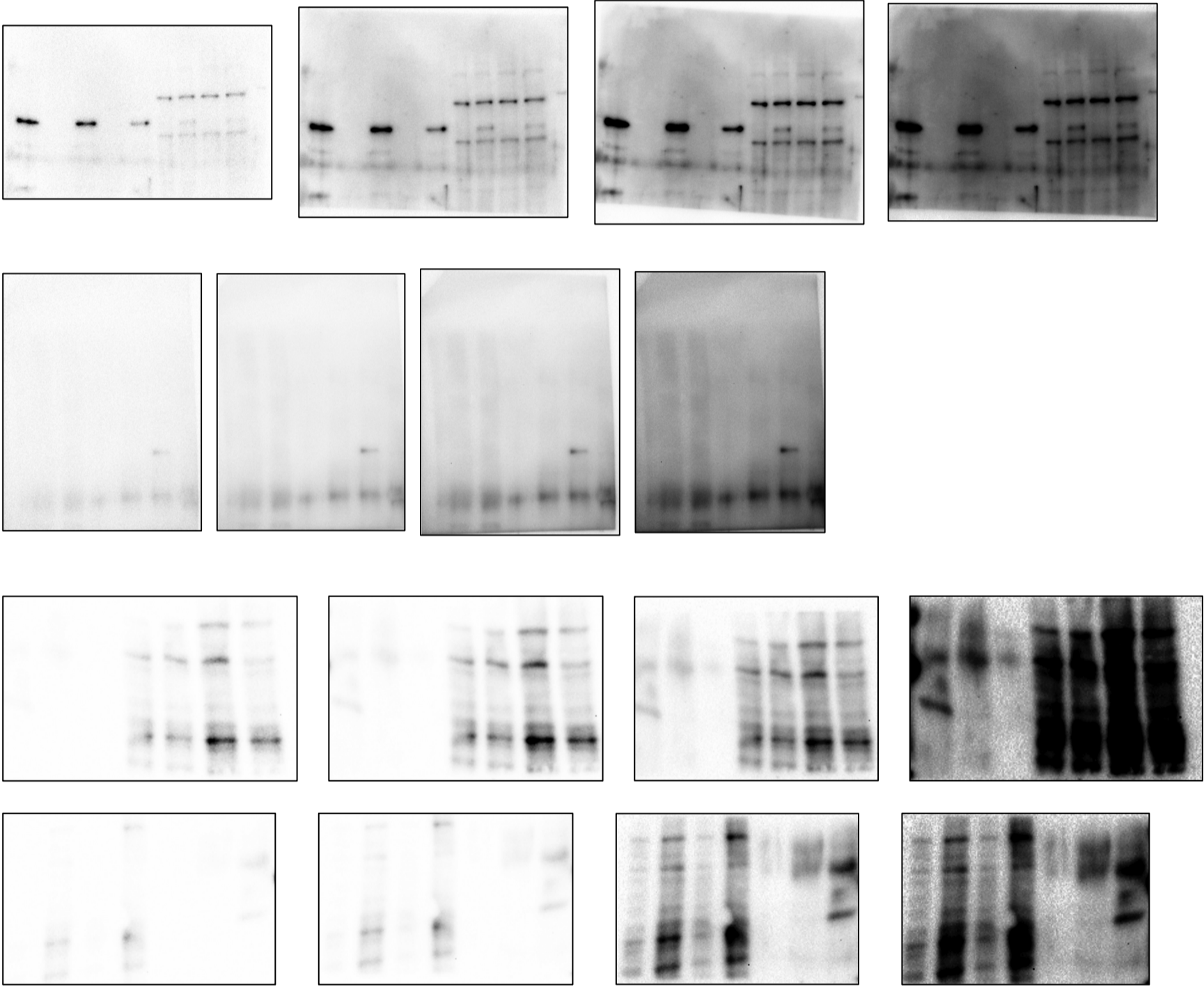

Fig. 5A

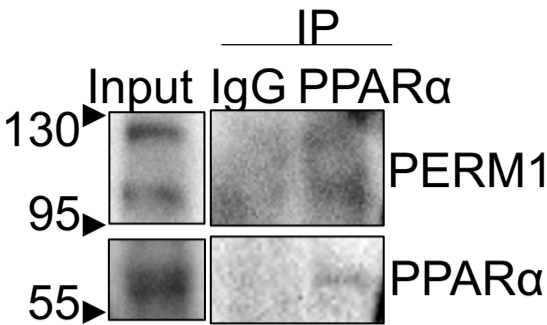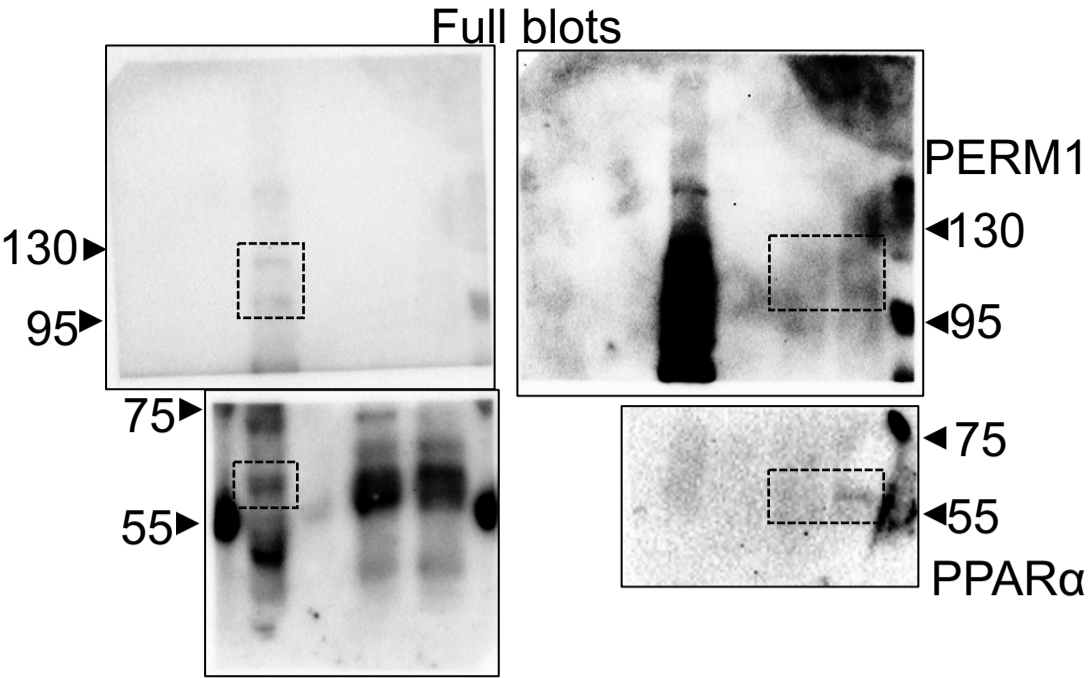

Multiple exposure images

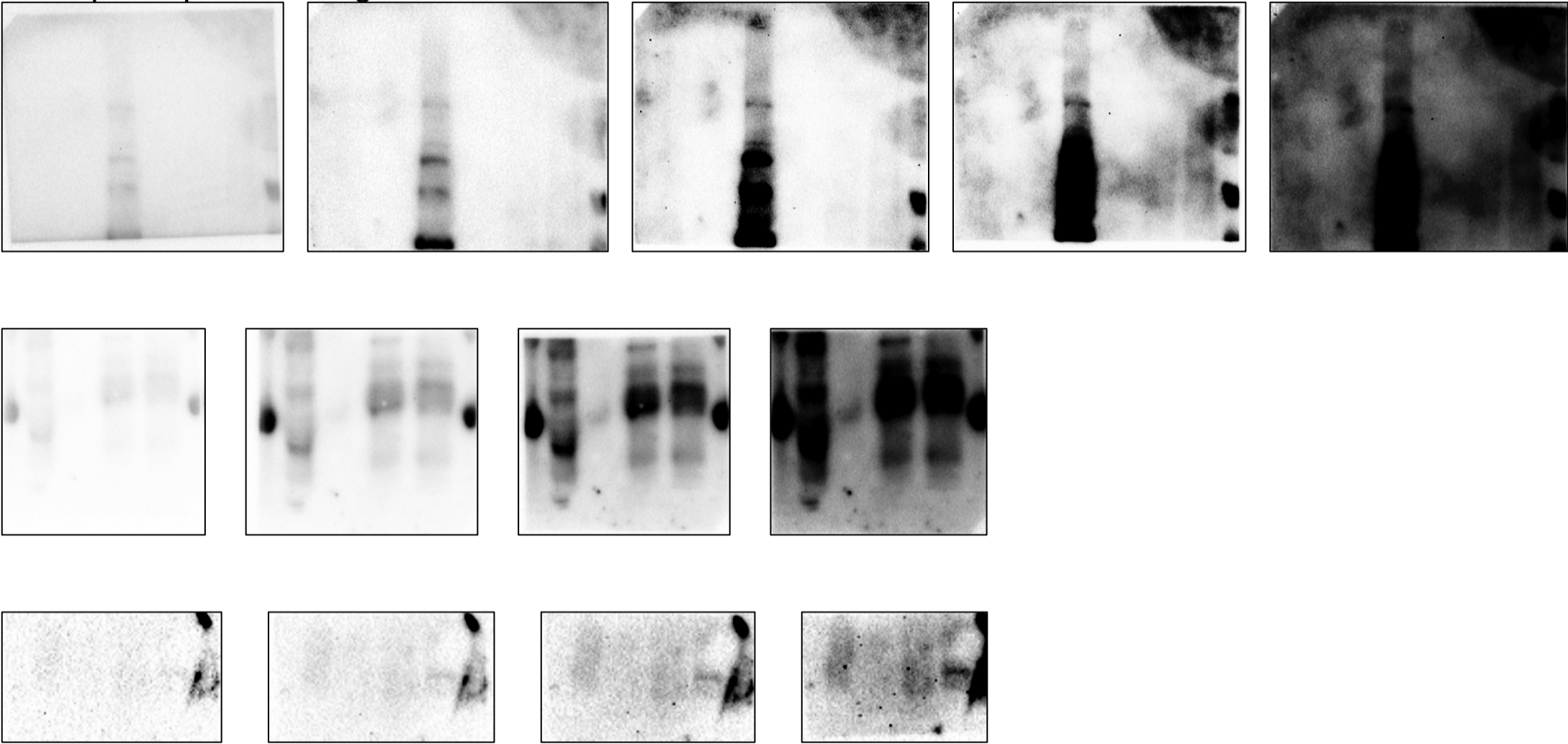

Fig. 5E

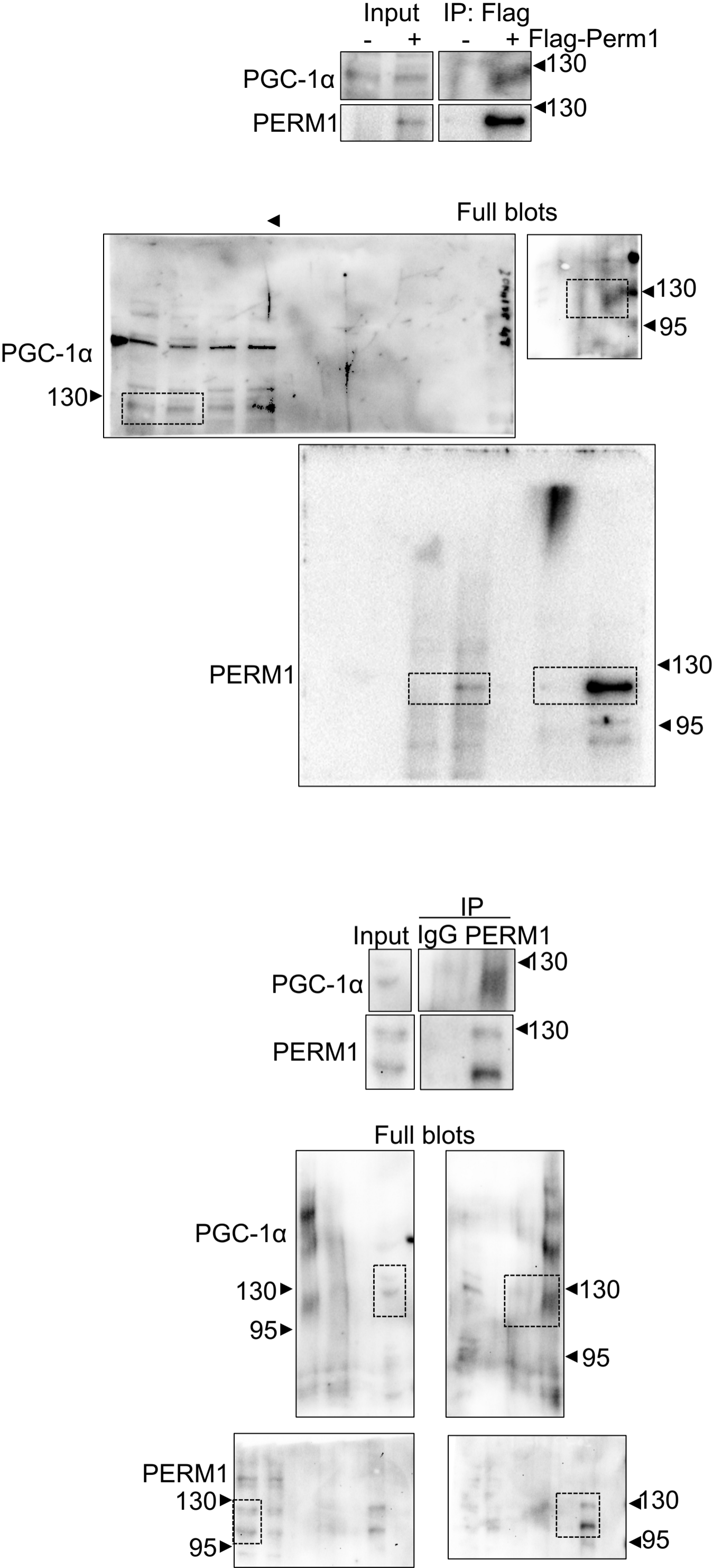

Fig. 5H

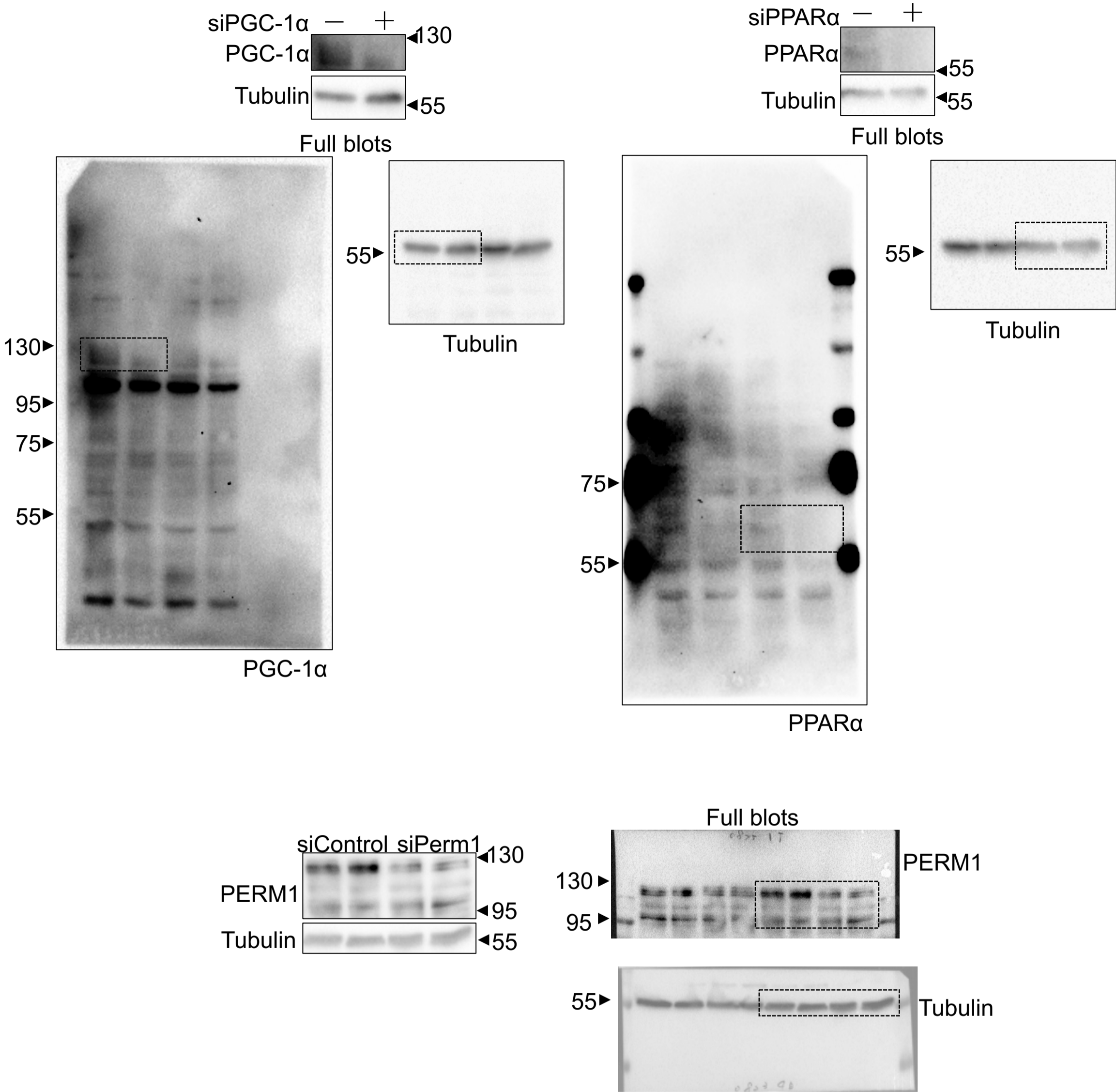

Supplement: Supplementary file 3 — Supplementary Information 3. [file 41598_2022_18885_MOESM3_ESM.pdf]
